# Peripheral Innate Lymphoid Cells Are Increased in First Line Metastatic Colorectal Carcinoma Patients: A Negative Correlation With Th1 Immune Responses

**DOI:** 10.3389/fimmu.2019.02121

**Published:** 2019-09-06

**Authors:** Romain Loyon, Marine Jary, Bérengère Salomé, Alejandra Gomez-Cadena, Jeanne Galaine, Marie Kroemer, Pedro Romero, Sara Trabanelli, Olivier Adotévi, Christophe Borg, Camilla Jandus

**Affiliations:** ^1^Department of Oncology UNIL CHUV, Ludwig Institute for Cancer Research, Lausanne Branch, University of Lausanne, Lausanne, Switzerland; ^2^Univ. Bourgogne Franche-Comté, INSERM, EFS BFC, UMR1098, Interactions Hôte-Greffon-Tumeur/Ingénierie Cellulaire et Génique, Besançon, France; ^3^Department of Medical Oncology, University Hospital of Besançon, Besançon, France; ^4^INSERM CIC-1431, Clinical Investigation Center in Biotherapy, University Hospital of Besançon, Besançon, France; ^5^Department of Pharmacy, University Hospital of Besançon, Besançon, France

**Keywords:** ILC, metastatic colorectal cancer, Th1, chemotherapy, immunomonitoring

## Abstract

Several distinct innate lymphoid cell (ILC) populations have been recently identified and shown to play a critical role in the immediate immune defense. In the context of tumors, there is evidence to support a dual role for ILCs with pro- or antitumor effects, depending on the ILC subset and the type of cancer. This ambivalent role has been particularly well-described in colorectal cancer models (CRC), but the presence and the evolution of ILCs in the peripheral blood of metastatic CRC (mCRC) patients have not yet been explored. Here, we investigated the distribution of ILC subsets in 96 mCRC patients who were prospectively included in the “Epitopes-CRC02” trial. Peripheral blood mononuclear cells (PBMCs) were analyzed by flow cytometry at metastatic diagnosis and after 3-months of treatment. The treatments consisted of Oxaliplatin-based chemotherapies for 76% of the patients or Folfiri (5FU, Irinotecan) chemotherapies for 14% of patients. Compared to healthy donors, the frequency of total ILCs was dramatically increased at metastatic diagnosis. CD56^+^ ILC1-like cells were expanded, whereas ILC2, NCR^−^ ILCP and NCR^+^ ILCP subsets were decreased. Combined analysis with the systemic anti-telomerase hTERT Th1 CD4 response revealed that patients with low anti-TERT Th1 CD4 responses had the highest frequencies of total ILCs at diagnosis. Of those, 91% had synchronous metastases, and their median progression-free survival was 7.43 months (vs. 9.17 months for the other patients). In these patients, ILC1 and ILC2 were significantly decreased, whereas CD56^+^ ILC1-like cells were significantly increased compared to patients with low frequency of total ILCs and high anti-TERT responses. After treatment, the NCR^+^ ILCP were further decreased irrespective of the chemotherapy regimen, whereas the balance between ILC1 and CD56^+^ ILC1-like cells was modulated mainly by the Folfiri regimen in favor of ILC1. Altogether our results describe the effects of different chemotherapies on ILCs in mCRC patients. We also establish for the first time a link between frequency of ILCs and anti-tumor CD4 T cell responses in cancer patients. Thus, our study supports an interest in monitoring ILCs during cancer therapy to possibly identify predictive biomarkers in mCRC.

## Introduction

Colorectal cancer (CRC) has been used as a model to demonstrate the role of the immune system in cancer, notably to establish the prognostic role of memory T cell infiltration and Th17 predominance (1). Fifteen percent of localized CRC patients present microsatellite instability (MSI), with a higher mutation load conferring a higher response to immunotherapy (2). The immune response is also involved in CRC microsatellite stability (MSS), for example in consensus molecular subtype (CMS) four CRC patients (3). Research at the metastatic stage is ongoing in order to better define the CRC-stage-linked immune responses. It was previously described that cytotoxic innate immune cells, so called conventional natural killer (NK) cells, were almost absent in human colorectal tumors, despite efficient T cell infiltration (4). In contrast, a recent study on 13 localized human CRC and 13 lung tumors showed that helper innate immune cells, the recently identified family of innate lymphoid cells (ILCs) are present in these tumors, at different levels (5).

Distinct helper ILC populations have been reported to play a critical role on the maintenance of tissue homeostasis and immediate immune host defense (6). ILCs exhibit transcription factor and cytokine profiles that phenocopy the three major T helper (Th) cell subsets: Th1, Th2, and Th17/22. Thus, ILCs are classified into three subgroups, ILC1, ILC2, and ILC3 (7–9). The ILC3 subgroup can be further subdivided into NCR^+^ ILC3 that express the natural cytotoxicity receptor (NCR) NKp46, and NCR^−^ ILC3, which include lymphoid tissue inducer (LTi) cells. In addition, an extensive analysis of ILC from human peripheral blood and tissue showed recently that ILCs with c-Kit^+^ phenotype, previously proposed to represent human ILC3, are enriched in multi-potent and uni-potent ILC precursors (ILCP) in the peripheral blood that can give rise to all know ILC subsets (10).

ILCs produce high amounts of different cytokines very early after infection and tissue damage, suggesting potential pro- and anti-oncogenic roles of these cells during the early phases of carcinogenesis. ILC1s represent a very early source of IFN-γ that is mainly associated with a protective role against tumors through the upregulation of MHC molecules (11–13), the induction of Th1 polarization, and the activation of macrophages (14). Recently, we identified CD56^+^ ILC1-like cells, an ILC population within the ILC1 compartment, which constitutively expresses CD56 and CD127, but lack c-Kit; similar to Stage 4b NK cells they lack KIR and CD16, but they express NKG2A. CD56^+^ ILC1-like cells possess cytotoxic potential and have anti-leukemic effects (Salome et al., in revision). Similarly, CD103^+^ cytotoxic intraepithelial ILC1-like cells were previously reported in localized colorectal tumors (5) and were shown to directly kill tumor cells in a perforin-dependent manner (15, 16). Both pro- and antitumor roles of ILC2 in cancer biology have been suggested. Indeed, we and others reported on the expansion of ILC2, which contributed to the suppression of antitumor responses via an IL-13/monocytic-myeloid derived suppressor cell (M-MDSC) axis (17–19). In CRC, high levels of the ILC2-activating alarmin IL-33 and the prototypic ILC2 cytokine IL-4 are associated with poor prognosis (20, 21). In addition to this protumorigenic role, ILC2 also produce IL-5, which induces the selective expansion of eosinophils (22) whose degranulation has been shown to improve prognosis in various types of cancer, including CRC (23). Furthermore, secretion of IL-13 by ILC2 may promote the migration of activated DCs to tumor-draining lymph nodes resulting in cytotoxic T cell activation (24). ILC3 have also been described to play a protective role in carcinogenesis by limiting tissue damage through the secretion of IL-22 in a model of chronic colitis (25). However, IL-22 producing CCR6^+^ ILC3 may increase the tumorigenic potential of colon cancer (26–28). NCR^−^ ILC3-derived IL-22 can act on colon epithelial cells to sustain tumor progression. Moreover, IL-17 was shown to inhibit tumor progression by acting on T cells (29) and to contribute to reduced tumor growth and metastasis in mice inoculated with a colon cancer cell line (26). Nevertheless, an IL-23-Th17 gene signature in resected CRC was associated with a worse prognosis and found to predict rapid progression to metastatic disease, which demonstrates the potential pro-tumorigenic role of ILC3 (30).

These observations suggest the involvement of ILCs in natural tumor immunity. Furthermore, while a putative role for ILCs in mediating the effect of chemo-immunotherapy was demonstrated in a mouse model of melanoma (31), there is limited data available on the impact of anti-cancer treatments on ILC frequency and phenotype in humans. A recent study on human ovarian cancer reported that an ILC3-like population that expresses IFN-γ and IL-22 suppressed the activation and proliferation of tumor-infiltrating lymphocytes (TIL) *ex vivo*, suggesting a potential benefit of depleting these cells before TIL-based immunotherapy (32). In addition, a link between the efficacy of immune checkpoint blockades and ILCs is currently under investigation because ILC2 cells have recently been shown to express PD-1 (33, 34). Moreover, growing evidence suggests that chemotherapy may also modulate ILC homeostasis, in particular by altering the composition of commensal microbiota (35).

In this study, we assessed the modulation of ILC subsets in parallel with antigen-specific CD4 T cell responses in a large cohort of metastatic CRC (mCRC) patients, at baseline and 3 months postchemotherapy. Since human telomerase (hTERT) is expressed in many cancer types and plays a crucial role in oncogenesis by providing proliferation, survival and anti-apoptotic signals necessary for tumor progression (36–38), we used hTERT as a hallmark of cancer and as a universal tumor antigen prototype (39–41). We monitored the systemic anti-hTERT Th1 CD4 response and explored the relationship between anti-hTERT responses and the ILC compartment. We report here that ILCs are increased in favor of ILC1 in the peripheral blood of chemotherapy-naïve mCRC patients, and that the frequency of ILCs is negatively correlated with the cancer-specific Th1 immune response. Moreover, we observed that chemotherapy regimens, especially Folfiri, act on specific ILC subsets without affecting the total ILC population in the peripheral blood.

## Materials and Methods

### Patients and Healthy Donors

**Figure 1:**
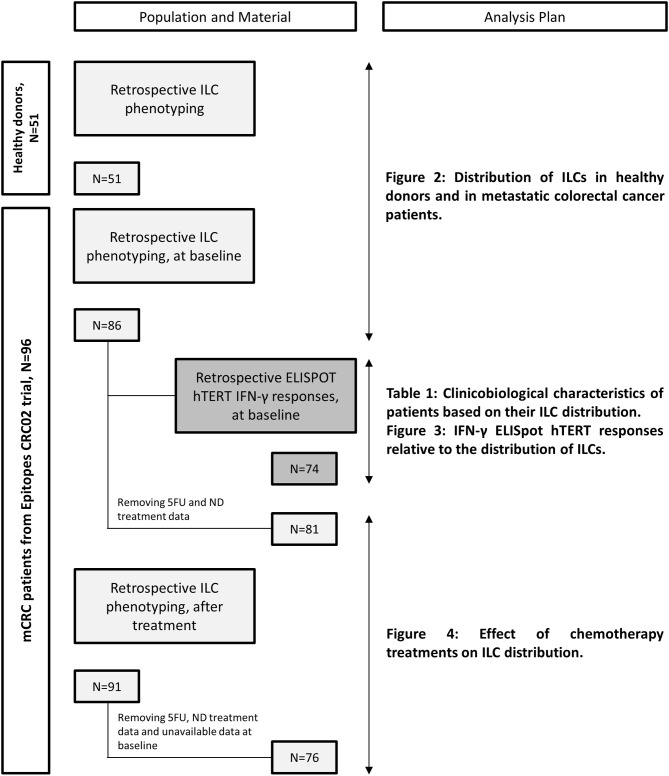
Flowchart of available materials and analysis plan.

Metastatic colorectal cancer patients were enrolled from March 2013 to August 2016 in the “Epitopes-CRC02” trial (NCT02817178), a French multicentric prospective study assessing the impact of treatment on the CD4 T cell response. All patients were enrolled after signing an informed consent, in accordance with French regulation, and after approval by local and national ethics committees. All data were anonymized. Overall population characteristics are summarized in Table S1. For each patient, blood samples were collected before any metastatic cancer specific treatment (baseline) and after 3 months of a chemotherapy-based regimen. The materials used and analysis plan are illustrated in Figure 1. Briefly, we monitored ILCs by flow cytometry in 86 patients at baseline and 76 matched patients after chemotherapy. From the samples collected after treatment, we retained data for patients receiving the following chemotherapy-based regimens: (A) 5FU and Irinotecan (Folfiri) (*n* = 19), (B) 5FU and Oxaliplatin (Folfox) (*n* = 40), and (C) 5FU, Oxaliplatin and Irinotecan (Folfoxiri) (*n* = 17). Patients treated with 5FU only, patients without treatment data available or patients without available data at baseline were removed from the analysis. Of note, we chose to include five patients who received Oxaliplatin and Raltitrexed, a folic acid analog that inhibits thymidylate synthetase similar to 5FU, in the Folfox branch. A total of 86% of patients received a bevacizumab regimen as well, regardless of the chemotherapy protocol. For healthy donor controls, blood cells were collected from anonymous donors at the *Etablissement Français du Sang* (Besançon, France) and the *Transfusion Interregionale* CRS (Lausanne, Switzerland) using an apheresis kit preparation, after obtaining signed informed consent.

### Cell Isolation

Peripheral blood mononuclear cells (PBMCs) were isolated from peripheral blood of patients and healthy donors by density centrifugation using a Ficoll gradient (GE healthcare). Cells were maintained in RPMI (Gibco) supplemented with 8% human serum, 100 U/ml penicillin-streptomycin (Invitrogen) and nonessential amino-acids.

### Antibodies

The following anti-human antibodies were used for the lineage the cocktail: CD3-FITC, CD4-FITC, CD8a-FITC, CD14-FITC, CD15-FITC, CD16-FITC, CD19-FITC, CD20-FITC, CD33-FITC, CD34-FITC, FcεRI-FITC, and CD203c-FITC (all from Biolegend). In addition, these anti-human antibodies were used: CD335 (NKp46)-PE/Cy7, CD294 (CRTH2)-PE, CD127-BV421 from Biolegend, CD56-APCeF780 from eBioscience, and CD117 (ckit)-APC from BD Bioscience. For intracellular transcription factor staining the following anti-human antibodies were used: Tbet-PE/CF594, GATA3-PE/Cy7, and RORγt-PE from BD Bioscience. Corresponding isotype control antibodies were used as controls.

### Staining and Flow Cytometry

To prepare for immunostaining, PBMCs were counted and suspended in FACS buffer (1X PBS, 50 μM EDTA, 0.2% BSA) prior to labeling with appropriate antibodies for 30 min in the dark at 4°C. The cells were then washed with 1X PBS. For the second step, cells were stained with the fixable viability dye, eFluor 506 (eBioscience), for 30 min at 4°C. The cells were then washed again with 1X PBS. Samples were evaluated on a FACS Canto II (BD Biosciences).

For transcription factor staining, after extracellular staining, cells were fixed and permeabilised using the InvitrogenTM eBioscience Foxp3/Transcription factor staining buffer Set according to manufacturer's recommendations. Intracellular antibodies were prepared in Permeabilisation buffer 1X and the intracellular staining was perform for 1 h at room temperature. After incubation, cells were washed with Permeabilisation buffer 1x twice and finally resuspended in FACs buffer. Samples were acquired using a Gallios II from Beckman Coulter.

Analysis were realized with FlowJo Software v 9.9.4. The gating strategy was set based on the isotype controls and applied in a standard format across all samples and all conditions.

### Assessment of Spontaneous hTERT-Specific CD4 T Cell Responses in Cancer Patients

An IFN-γ ELISpot was conducted for 74 patients of the cohort at baseline, as previously described by Godet et al. (42). Briefly, spontaneous responses were assessed by IFN-γ ELISpot after an *in vitro* stimulation of PBMCs with a mixture of eight peptides derived from hTERT (TERT_44−58_, TERT_578−592_, TERT_921−935_, TERT_1055−1069_, TERT_541−555_, TERT_573−587_, TERT_613−627_, and TERT_911−925_) at 5 μg/ml for 10 days. Another peptide mixture, referred to as CEF (PANATecs), derived from influenza virus (Flu), Epstein Barr virus (EBV), and cytomegalovirus (CMV) was used to evaluate antiviral recall responses in the same patients. CEF IFN-γ ELISpot data were available for 69 of the 74 patients. After the 10-day incubation with the peptide mixtures, the cells (10^5^ per well) were cultured in triplicate in the ELISpot plate with restricted peptides mixture or individual peptides at 5 μg/mL in X-vivo 15 medium (Ozyme, BE04–418) for 15 h. The IFN-γ spots were developed following the manufacturer's instructions (Diaclone, 856 051 020P). Spot-forming cells were counted using the C.T.L. Immunospot system (Cellular Technology Ltd.). The number of specific T cells expressed as the number of spot-forming cells per 10^5^ cells was calculated after subtracting the negative control values (background). Responses were considered positive when the number of IFN-γ spots was >10 and greater than twice the background. Responses with a background >100 spots were excluded from the analysis.

### Statistical Analysis

Numerical data are expressed as the mean ± SEM (Standard Error Mean). Student's *t*-tests, ANOVAs and Chi-square tests were used for the evaluation of statistical significance, calculated with GraphPad Prism 8.0 or R Studio. *P*-values lower than 0.05 were considered significant with *p < 0.05, **p < 0.005, ***p < 0.001, and ****p < 0.0001. Progression free survival (PFS) was calculated from the date of study enrolment to the date of tumor progression. Kaplan-Meier survival curves were generated using R Studio® software (median with 95% confidence interval).

## Results

### Chemotherapy-Naïve Metastatic Colorectal Cancer Patients Have Increased Frequencies of ILCs and Their Subsets' Distribution Is Skewed Toward ILC1

**Figure 2:**
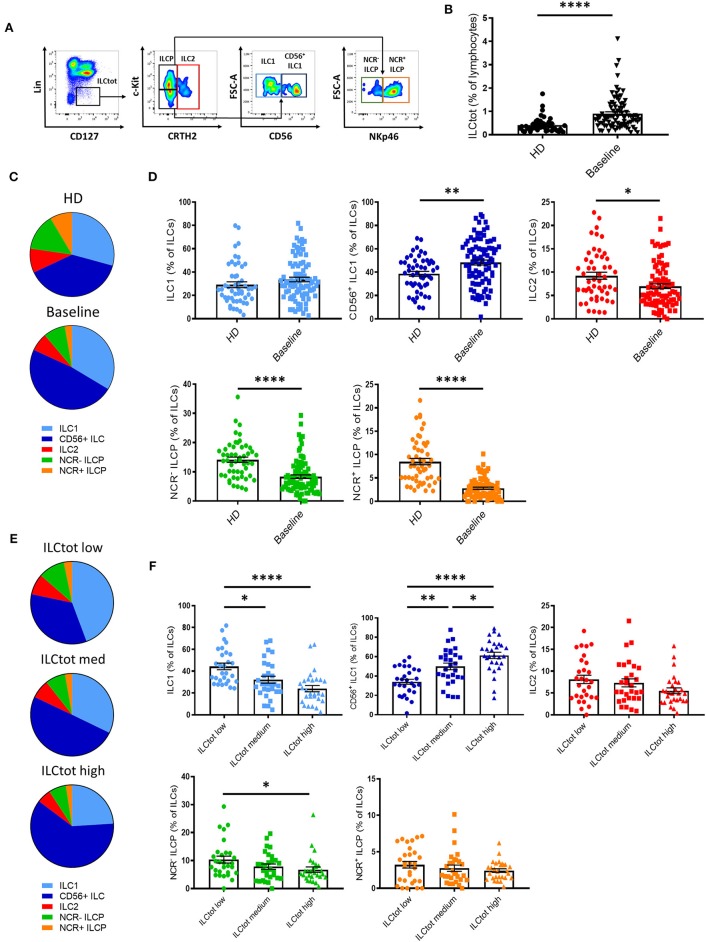
Distribution of ILCs in healthy donors and in metastatic colorectal cancer patients. **(A)** Flow cytometry gating strategy for ILC subset identifications in PBMCs. **(B)** Frequencies of ILCtot. ILC subset distributions **(C)** and frequencies **(D)** were analyzed by flow cytometry in PBMCs of 51 healthy donors (HD) and 86 chemotherapy-naïve metastatic colorectal cancer patients (baseline). Distribution **(E)** and frequencies **(F)** of ILC subsets based on the frequency of ILCtot distributed in the low (*n* = 29), medium (*n* = 29), and high (*n* = 28) terciles. Columns, the means of ILC frequency for each patient; bars, SEM. *p < 0.05, **p < 0.005, ****p < 0.0001, as determined by Student's *t*-test or ANOVA.

To evaluate the impact of mCRC on ILCs, we investigated ILC frequency and subset distribution in PBMCs of 86 chemotherapy naïve (baseline) mCRC patients included in the “Epitope-CRC02” study (Table S1). Fifty-one healthy donors (HD) were used as a control cohort. The percentage of total ILCs (ILCtot) (Lin^−^ CD127^+^) was assessed by flow cytometry, as well as the five ILC subsets, defined as ILC1 (Lin^−^ CD127^+^ cKit^−^ CRTH2^−^ CD56^−^), CD56^+^ ILC1-like (Lin^−^ CD127^+^ cKit^−^ CRTH2^−^ CD56^+^), ILC2 (Lin^−^ CD127^+^ CRTH2^+^), NCR^−^ ILCP (Lin^−^ CD127^+^ cKit^+^ NKp46^−^), and NCR^+^ ILCP (Lin^−^ CD127^+^ cKit^+^ NKp46^+^) (Figure 2A and Figure S1A). In addition, we validated our gating strategy by assessing master transcription factor expression of each population by flow cytometry (Figure S1B). For ILC2, functionally distinct c-Kit^+^ and c-Kit^−^ ILC2 have recently been described in healthy donors (43). Interestingly, in mCRC patients ILC2 were almost exclusively c-Kit^−^ (Figure S2). Thus, in subsequent analysis, ILC2 were analysis only based on the expression of CRTH2. We observed that the frequency of ILCtot are significantly increased in the PBMCs of mCRC patients at baseline compared to HD (Figure 2B). Moreover, the distribution of the ILC subsets was also distinct in chemotherapy-naïve mCRC patients (Figures 2C,D). The ILC2, NCR^+^ ILCP and NCR^−^ ILCP subsets were significantly decreased in patients at baseline compared to HD, whereas the ILC1 and CD56^+^ ILC1-like subsets were increased. We next stratified the patients according to their total ILC tercile distribution: ILCtot low [0.09–0.54%], *n* = 29; ILCtot medium [0.54–0.96%], *n* = 29; and ILCtot high [0.99–4.11], *n* = 28. For each group, the ILC subset distribution was analyzed, and the results showed that the ILC subset proportions are linearly modulated (Figures 2E,F). Deeper analysis revealed that the ILC1 population proportion was significantly decreased in ILCtot high patients, whereas CD56^+^ ILC1-like cell proportion was increased. Of note, ILCtot low patients had the highest proportion of NCR^−^ ILCP and NCR^+^ ILCP cells. Overall, these results show that ILCs are drastically increased in the peripheral blood, with a skewing toward ILC1s at CRC metastatic diagnosis. Moreover, patients with high proportions of ILCtot present the lowest rates of ILC1 and NCR^−^ ILCP cells in favor of CD56^+^ ILC1-like cells.

### Patient Clinicobiological Characteristics and ILC Distribution

To evaluate the link between ILCs and clinicobiological data we focused our analysis on 74 patients for which ILC frequencies and tumor-specific Th1 immunity (focusing on hTERT as a universal tumor-antigen) were available. The clinicobiological characteristics available for these patients are summarized in Table 1. Because we observed that ILCtot high patients presented at least 2-fold more ILCs than HD, for clinical correlations we decided to perform our analyses with merged ILCtot medium and ILCtot low patients (referred to hereafter as ILCtot low) (Table 1). ILCtot high patients did not differ from the rest of the cohort with regards to the age, gender, tumor location, time of metastases or metastatic location. Regarding biological and molecular parameters, no difference was observed between the two groups. Of note, ILC counts were not influenced by microsatellite status, RAS and BRAF mutations, carcinoembryonic antigen (CEA) value or lymphocyte count. Interestingly, only 13% of MSI tumors had an ILCtot high phenotype and the BRAF mutation prognostic biomarker was not enriched in the ILCtot high patients. Tumor response rate was also not different based on the profile of ILC distribution.

**Table 1:** Clinicobiological characteristics of patients based on their ILC distribution.

	**IFN-γ ELISpots hTERT patients at baseline**	**ILC low**	**ILC high**	***p***
	*n* = 74 (%)	*n* = 50 (%)	*n* = 24(%)	
**AGE-YEARS**				
<65	*n* = 36 (49%)	22 (44%)	14 (61%)	
≥65	*n* = 37 (51%)	28 (56%)	9 (49%)	0.28
**GENDER**				
F	*n* = 27 (36%)	16 (32%)	11 (46%)	
M	*n* = 47 (64%)	34 (68%)	13 (54%)	0.37
**TUMOR LOCATION**				
Rectum	*n* = 19 (27%)	14 (30%)	5 (21%)	
Colon	*n* = 52 (73%)	33 (70%)	19 (79%)	0.60
**MICROSATELLITES**				
MSI	*n* = 8 (21%)	7 (28%)	1 (7%)	
MSS	*n* = 31 (79%)	18 (72%)	13 (93%)	0.26
**RAS STATUS**				
M	*n* = 33 (50%)	20 (45%)	13 (59%)	
WT	*n* = 33 (50%)	24 (55%)	9 (41%)	0.43
**BRAF STATUS**				
M	*n* = 8 (14%)	5 (11%)	3 (14%)	
WT	*n* = 58 (86%)	39 (89%)	19 (86%)	0.99
**TIME OF METASTASIS**				
Metachronous	*n* = 14 (20%)	12 (24%)	2 (8%)	
Synchronous	*n* = 57 (80%)	35 (76%)	22 (92%)	0.16
**METASTATIC LOCALIZATION**				
Extra hepatic	*n* = 16 (22%)	*n* = 14 (29%)	*n* = 2 (8%)	
Hepatic and other	*n* = 19 (26%)	*n* = 12 (25%)	*n* = 7 (29%)	
Hepatic only	*n* = 37 (52%)	*n* = 22 (46%)	*n* = 15 (63%)	0.13
**METASTATIC LOCALIZATION**				
Extra hepatic/hepatic and other	*n* = 35 (49%)	*n* = 26 (54%)	*n* = 9 (37.5%)	
Hepatic only	*n* = 37 (51%)	*n* = 22 (46%)	*n* = 15 (62.5%)	0.28
**LYMPHOCYTE COUNT**				
<1,000	*n* = 9 (13%)	5 (11%)	4 (17%)	
≥1,000	*n* = 61 (87%)	41 (89%)	20 (83%)	0.75
**ACE**				
<20	*n* = 26 (43%)	19 (46%)	7 (35%)	
≥20	*n* = 35 (57%)	22 (54%)	13 (65%)	0.57
**TUMOR RESPONSE**				
Progression disease	*n* = 4 (7%)	2 (5%)	2 (10%)	
Stable disease	*n* = 21 (36%)	13 (35%)	8 (40%)	
Partial response	*n* = 29 (50%)	21 (55%)	8 (40%)	
Complete response	*n* = 4 (7%)	2 (5%)	2 (10%)	0.66

*The ILCtot frequencies of patients at baseline were distributed into terciles. The results shown for the low (n = 25) and medium (n = 25) terciles were pooled and referred to as ILC low (n = 25 + 25 = 50), and the high tercile was referred to as ILC high (n = 24). ELISpot hTERT response data was available for all 74 patients, however, data for some clinicobiological characteristics are missing for some patients*.

Next, some hypotheses were formulated regarding the different subsets of ILCs and were subsequently tested with our data. (i) We first hypothesized that MSI CRC patients harbor a higher mutational load and an interferon gamma (IFN-γ) signature. However, we observed that ILC subsets were distributed equally in the nine MSI tumors (data not shown), with the ILC1 subset count higher in only two patients. (ii) Next, inflammatory contextures, such as mucosal immunity or hepatic metastatic localization, were specifically assessed for ILC subsets. In the 68 synchronic patients with a primary colon tumor in place, the ILC subset proportions were largely the same. The ILC subset proportions were almost equally distributed among each different subset. (iii) We then hypothesized that cytokine availability following lymphopenia could enhance the number of ILCs, as has been described for NK cells after transplantation (44, 45). However, here we did not observe such an expansion, regardless of which ILC subset was considered. (iv) BRAF mutations are present in 5–8% of mCRC patients and are often associated with MSI tumors, however, less is known about BRAF mutations in MSS tumors. Of the nine BRAF mutated patients in our cohort, three had an MSS phenotype, however, none of these patients presented any specific enrichment for one ILC subset.

### ILCs Are Negatively Correlated With the Th1 Anti-hTERT Immune Response

**Figure 3:**
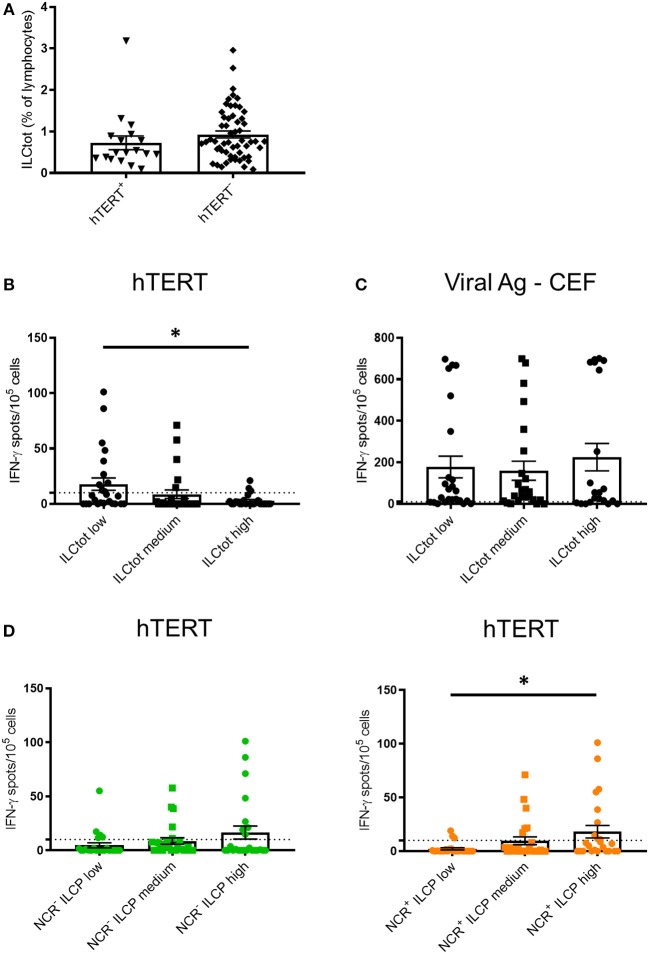
IFN-γ ELISpot hTERT responses relative to the distribution of ILCs. PBMCs from 74 chemotherapy-naïve metastatic colorectal cancer patients were cultured with a mixture of 8 hTERT peptides (TERT_44−58_, TERT_578−592_, TERT_921−935_, TERT_1055−1069_, TERT_541−555_, TERT_573−587_, TERT_613−627_, and TERT_911−925_) at 5 μg/mL. The T cell reactivity against the hTERT peptides was detected by IFN-γ ELISpot assays, as described in the Material and Methods. Stimulation with Viral Ag-CEF peptides was used as control for T cell reactivity. **(A)** The frequency of ILCtot in the PBMCs of each patient was analyzed based on the positive (*n* = 18) or negative (*n* = 56) response to hTERT. **(B,C)** Results are shown as the means of IFN-γ spot numbers (magnitude) and the ILC frequencies (ILCtot distributed into low (*n* = 25), medium (*n* = 25) and high (*n* = 24) terciles) for hTERT responses **(B)** and Viral Ag-CEF responses **(C)**. **(D)** Results are shown as the means of IFN-γ spot numbers (magnitude) and frequencies of NCR^−^ ILCP (left) and NCR^+^ ILCP (right) distributed among the low (*n* = 25), medium (*n* = 25) and high (*n* = 24) terciles. Columns, the means of spots from triplicate wells; bars, SEM. *p < 0.05, as determined by Student's *t*-test.

To explore the relationship between the adaptive immune system and ILCs, we took advantage of promiscuous hTERT-derived MHC class II peptides that allow for monitoring of anti-telomerase Th1 CD4 immunity in most HLA contexts (42, 46, 47). For this purpose, IFN-γ production by PBMCs derived from 74 chemotherapy naïve mCRC patients exposed to hTERT promiscuous peptides was measured by ELISpot in short-term stimulation assays. hTERT-derived peptides were recognized by PBMCs from 18 of the patients analyzed (24.3%) (Figure 3A). A trend of lower frequency of ILCs was observed in these patients in comparison to the mCRC patients who had no Th1 responses after hTERT-peptide stimulation. For each ILCtot group, the number of IFN-γ spots after MHC class II restricted hTERT peptide stimulation were analyzed (Figure 3B). Interestingly, ILCtot low patients had a higher anti-telomerase Th1 response compared to patients with high ILC levels. As a comparison to the cancer antigen hTERT, we concurrently measured T cell reactivity against a mixture of viral peptides in the same patient group (Figure 3C). Anti-viral T cell responses were detected in most patients (76.8%). Unlike the systemic anti-hTERT Th1 response, the frequency of patients exhibiting antiviral T cell responses was equally distributed among the patient subgroups. These results suggest that the presence of circulating anti-hTERT Th1 responses is related to the ILCtot proportion. Interestingly, regardless of the ILCtot percentage, patients with the highest proportion of ILCP had a better Th1 response compared to patients with the lowest NCR^+/−^ ILCP proportion (Figure 3D).

To explore the prognostic value of ILCtot high patients having lower anti-hTERT Th1 responses, we estimated the PFS of the ILCtot high and ILCtot low patients (Figure S3). In our cohort, the median of PFS was 7.43 months (95% CI: 6.44–14) for the ILCtot high patients, and 9.17 months (95% CI: 7.62–11.8) for the ILCtot low patients (*p* = 0.3). A better prognosis tendency was observed for the ILCtot low patients in our cohort, especially after 15 months.

Our results show that the frequency of ILCtot in the peripheral blood of mCRC patients at baseline is negatively correlated with anti-hTERT Th1 immune responses.

### Chemotherapy Treatment of mCRC Modulates ILC Subsets' Distribution

**Figure 4:**
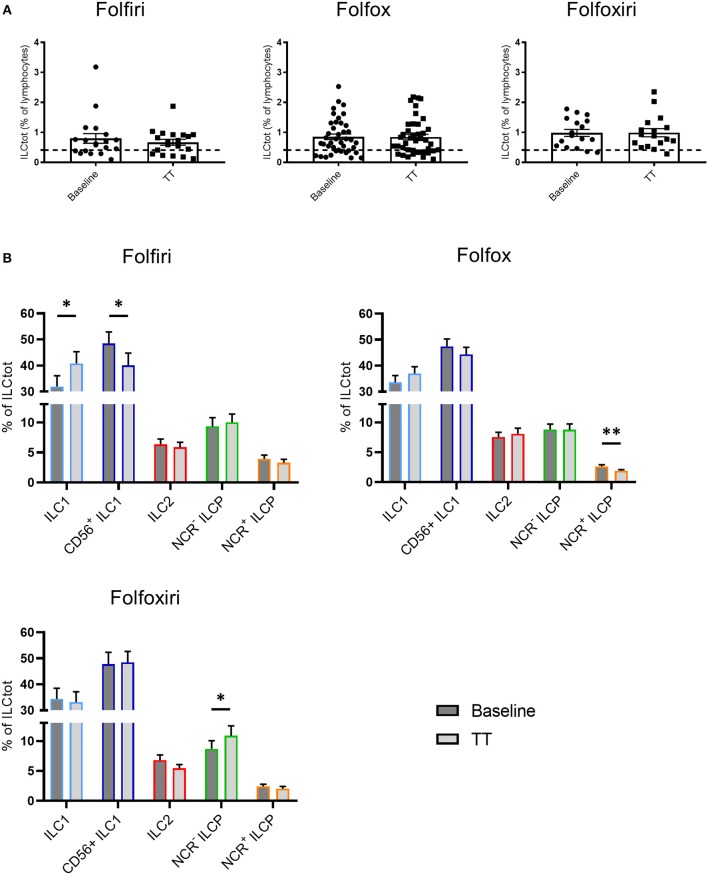
Effect of chemotherapy treatments on ILC distribution. Frequencies of ILCtot **(A)** and ILC subsets **(B)** were analyzed by flow cytometry in PBMCs from chemotherapy-naïve mCRC patients (baseline) and after 3 months of treatment (TT). The mean frequency of ILCtot for HD is represented by a dashed line. Each patient received either the 5FU/Irinotecan regimen (Folfiri) (baseline/TT matched patients, *n* = 19), the 5FU/Oxaliplatin regimen (Folfox) (baseline/TT matched patients, *n* = 40), or the 5FU/Oxaliplatin/Irinotecan regimen (Folfoxiri) (baseline/TT matched patients *n* = 17). *p < 0.05, **p < 0.005, as determined by Student's *t*-test.

Most conventional chemotherapies present immunogenic properties that at least partially contribute to their clinical efficacy (48). To evaluate the impact of chemotherapies on ILCs, we performed a comparative analysis of total and ILC subset frequency in peripheral blood of chemotherapy naïve mCRC patients at baseline and on the same patients after 3 months of treatment (TT in Figure 4) with the classical chemotherapy regimens, Folfiri (5FU and Irinotecan; *n* = 19), Folfox (5FU and Oxaliplatin; *n* = 40), and Folfoxiri (5FU, Oxaliplatin and Irinotecan; *n* = 17). The results showed that chemotherapies did not affect the frequency of total ILCs, which remain high after treatment (Figure 4). However, chemotherapies modulated the distribution of ILC subsets. The Folfiri regimen significantly reversed the balance between ILC1 and CD56^+^ ILC1-like cells, while ILC2 were not affected by any of the treatments. All of the regimens induced a decrease in NCR^+^ ILCP that reached significance in the Folfox group, whereas NCR^−^ ILCP were significantly increased in the Folfoxiri group. Collectively, these data highlight the ILC modulation potential of chemotherapy treatments.

## Discussion

ILC dysregulation in terms of frequency and subset composition has already been described in hematological malignancies and some solid tumors (18, 19, 49–51). Here we analyzed ILC populations in mCRC patients pre- and postchemotherapy. We showed that ILCtot are drastically increased in the peripheral blood, suggesting cytokine stimulation in the ILC compartment. IL-7, largely described as a key stimulator of ILC proliferation and survival (52), could play such a role. Indeed, IL-7 was recently described as particularly elevated in the serum of CRC patients, especially in patients with metastases (53). Deeper analysis revealed that the distribution of ILC subsets was altered in CRC patients. Indeed, only ILC1 and CD56^+^ ILC1-like cells were increased in mCRC patients at baseline compared to HD, whereas ILC2 and especially NCR^−^ ILCP and NCR^+^ ILCP were decreased. Recently, increased TGF-β in the tumor microenvironment was described as a key factor driving immune evasion and metastases in CRC (54). Interestingly, the conversion of NK cells into noncytotoxic ILC1 has been reported as a novel mechanism of tumor immune escape, being dependent on TGF-β (55, 56). Indeed, the plasticity of ILCs could impact their role in cancer development. Collectively, these data suggest that ILC1 and ILC1-like cells could be increased due to TGF-β in mCRC patients. Further investigations are necessary to understand if the plasticity of ILCs is involved in this conversion or if the proliferation of CD56^+^ ILC1-like cells is specifically favored.

Immunity against cancer is mainly mediated by Th1 cells (57, 58). Th1 immune infiltration is correlated to a better prognosis for patients, notably in CRC. The molecular analysis of cytotoxic and helper T cell populations in 125 colorectal tumor specimens by Galon et al. (30) showed that patients with high Th1 cluster expression have prolonged disease-free survival. More recently, data suggested that circulating tumor-specific T cells might reflect *in situ* events in the tumor (59, 60), since cancer immunity is a dynamic process that involves cell trafficking through the peripheral blood. Our results showed that higher systemic IFN-γ Th1 responses correlated with a lower frequency of total ILCs, but higher proportions of ILCP subsets. Thus, IFN-γ produced by ILC1 could drive the adaptive response toward a cancer specific Th1 profile. ILCP cells seem to also be associated with the Th1 immune response because the highest proportions of ILCP cells were found in patients with the strongest Th1-derived IFN-γ responses. It has been recently reported that NCR^+^ ILCP cells are increased in human non-small cell lung cancer (NSCLC) tissue and might contribute to the formation of protective tumor-associated tertiary lymphoid structures (61). These observations might explain the specific increase in anti-tumor CD4 T cell responses.

Even if no significant PFS differences could be observed when comparing ILCtot low vs. high patients, there was a tendency for higher PFS in ILCtot low patients with high Th1 hTERT-related responses. These observations are in line with previous studies where spontaneous universal cancer peptide (UCP)-specific T-cell immune responses correlated with increased overall survival of patients responding to chemotherapy in NSCLC and anal squamous cell carcinoma (42, 62). Interestingly, no significant differences were observed between each group regarding the clinicobiological characteristics. However, it is interesting to note that the ILCtot high group was composed of only 13% MSI tumors, even if these observations are based on a few patients and considering that MSI tumors are rare in metastatic setting. In addition, MSI is known to increase the T cell repertoire involved in tumor immunity conferring a higher response to immunotherapy (2).

The immune contexture determined at diagnosis influences the prognosis of cancer patients, including patients with CRC (63). Moreover, it turned out that widely used conventional chemotherapies modulated the composition and functionality of tumor immune infiltrates and this affected disease outcome (48, 64). In addition, even if chemotherapy causes massive lymphodepletion, this could ultimately reset the immune system by favoring a rebound replenishment of various immune cell subsets and/or allow the emergence of a specific effector cell type with anticancer activity (65, 66). In this study, we explored the effects of chemotherapy regimens on the ILC compartment and we showed that the balance between NCR^+^ ILCP and NCR^−^ ILCP was modulated. Indeed, NCR^+^ ILC3 were decreased with all regimens, and especially with Folfox, whereas NCR^−^ ILCP were increased especially with Folfoxiri. Both regimens include Oxaliplatin, an agent already described to be an activator of tumor-targeting immune responses, mainly through the induction of immunogenic cell death (67). Folfiri does not include Oxaliplatin and is less commonly described as an immunologic modulator. However, Folfiri regimens induced a decrease in CD56^+^ ILC1-like cells in favor of ILC1 cells. Interestingly, Folfiri has been described to support the expansion of circulating myeloid-derived suppressor cells (MDSCs), while this is not the case for Folfox (68). Thus, it would be interesting to explore the relationship between ILC1/ILC1-like cells and MDSCs in the context of mCRC.

To conclude, this study showed that ILCs are increased in the peripheral blood of chemotherapy-naïve mCRC patients and that their subset distribution is modulated. Moreover, the frequency of ILCs was found to be negatively correlated with the cancer-specific Th1 immune response. Finally, we showed that chemotherapy regimens act on the ILC compartment, especially Folfiri treatment, which modulated the balance between ILC1 and ILC1-like cells. Altogether, these results highlight the importance of considering the ILC compartment in the monitoring of immune responses in cancer to better define immune scores and eventually to identify useful biomarkers.

## Ethics Statement

This study was carried out in accordance with the recommendations of ANSM (*Agence Nationale de Sécurité du Médicament et des produits de santé*; no. 2012-A01377-36) with written informed consent obtained from all subjects in accordance with the Declaration of Helsinki. The protocol was approved by the independent Est-II French Committee for the Protection of Persons no. 12/672.

## Author Contributions

RL, BS, AG-C, and ST conducted the experiment. RL and MJ carried out data analysis. RL, MJ, CB, and CJ designed the research study. RL, MJ, BS, JG, MK, PR, ST, OA, CB, and CJ discussed the results, and wrote and/or reviewed the manuscript.

### Conflict of Interest Statement

The authors declare that the research was conducted in the absence of any commercial or financial relationships that could be construed as a potential conflict of interest.

## Supplementary Material

Figure S1 Flow cytometry gating strategy and expression of transcription factors for human ILC subsets. **(A)** Flow cytometry gating strategy for ILC subset identifications in PBMCs of a representative HD and a representative mCRC patient. **(B)** Transcription factors T-bet, GATA3 and RORγt were assessed by intracellular staining respectively on ILC1 and CD56^+^ ILC1, ILC2, and ILCP. Representative example of the staining on PBMCs of a healthy donor. Gray histograms correspond to the FMO.

Figure S2 Distribution of ILC2 c-Kit^−^ and c-Kit^+^ in healthy donors and metastatic colorectal cancer patients. Percentage of ILC2 c-Kit^−^ and c-Kit^+^ for HD and metastatic colorectal cancer patients at baseline and after treatment (TT). Columns, the means of ILC2 for each patient; bars, SEM. *****p* < 0.0001, NS (non-significant) as determined by Student's *t*-test.

Figure S3 Progression-Free Survival Kaplan-Meier curves of chemotherapy-naïve metastatic colorectal cancer patients. Patients (*n* = 73) stratified by the frequency of total ILCs distributed into low + medium (*n* = 50) vs. high (*n* = 23) terciles.

Table S1 Clinicobiological characteristics of CRC patients. The table summarizes the clinicobiological characteristics of CRC patients enrolled in the “Epitopes-CRC02” trial (NCT02817178) before (baseline) and after chemotherapy treatment (*n* = 96).
