# Supplementary figures and images for: Peripheral Innate Lymphoid Cells Are Increased in First Line Metastatic Colorectal Carcinoma Patients: A Negative Correlation With Th1 Immune Responses
Source: Front Immunol. 2019 Sep 6;10:2121. doi: 10.3389/fimmu.2019.02121 (PMC6742701; doi:10.3389/fimmu.2019.02121)

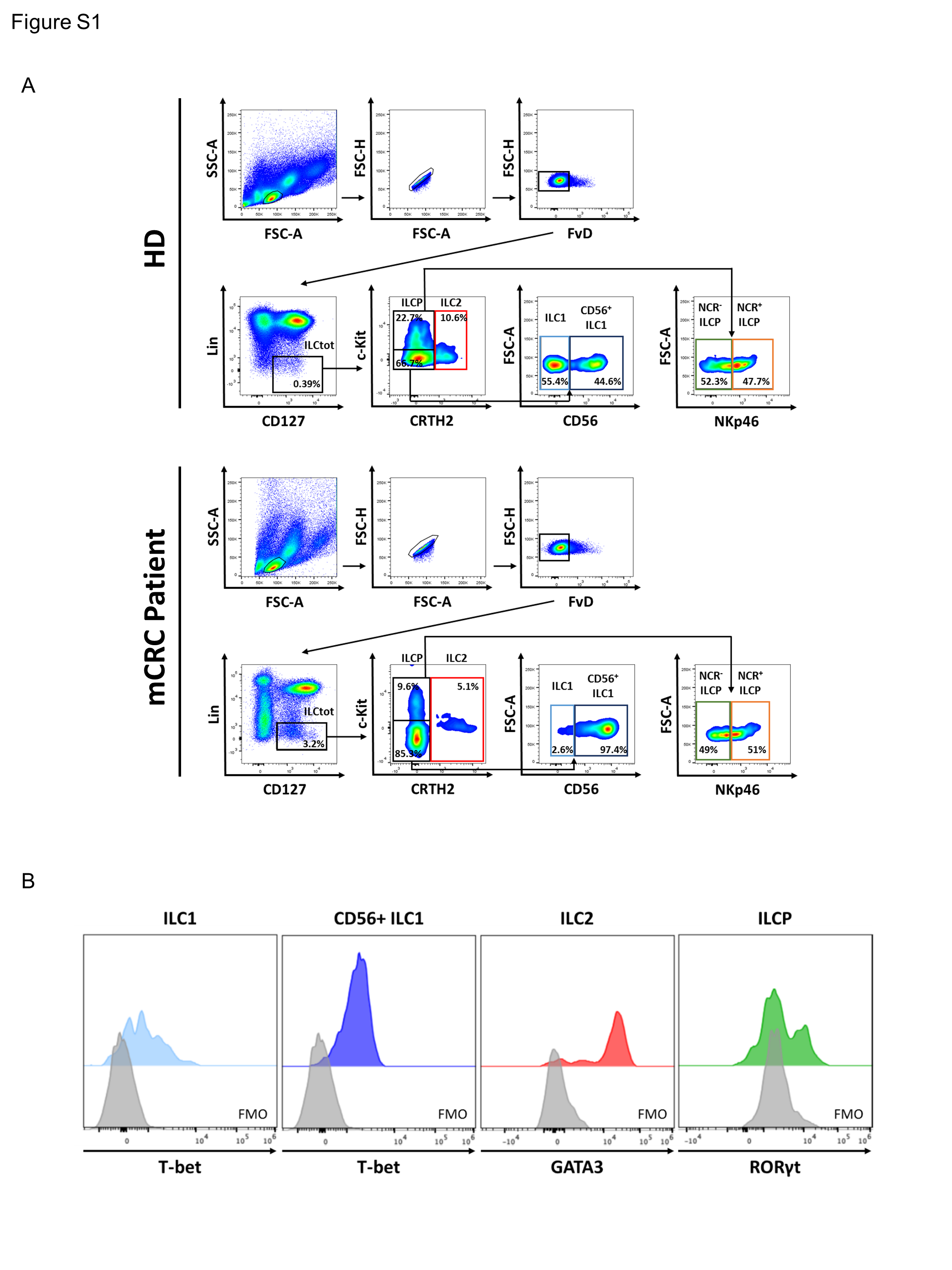

Supplement: Figure S1 — Flow cytometry gating strategy and expression of transcription factors for human ILC subsets. (A) Flow cytometry gating strategy for ILC subset identifications in PBMCs of a representative HD and a representative mCRC patient. (B) Transcription factors T-bet, GATA3 and RORγt were assessed by intracellular staining respectively on ILC1 and CD56+ ILC1, ILC2, and ILCP. Representative example of the staining on PBMCs of a healthy donor. Gray histograms correspond to the FMO. [file Image_1.tif]

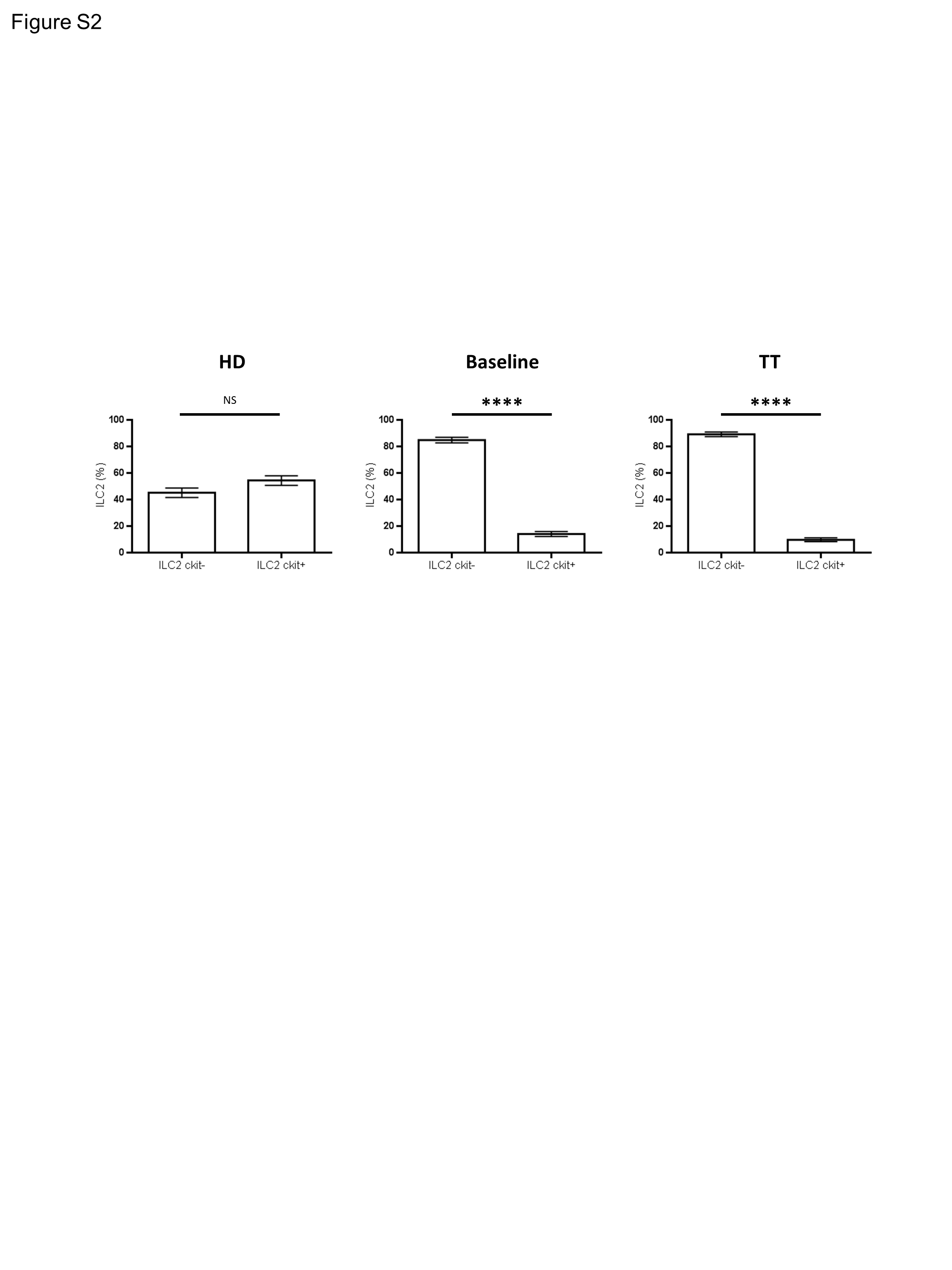

Supplement: Figure S2 — Distribution of ILC2 c-Kit− and c-Kit+ in healthy donors and metastatic colorectal cancer patients. Percentage of ILC2 c-Kit− and c-Kit+ for HD and metastatic colorectal cancer patients at baseline and after treatment (TT). Columns, the means of ILC2 for each patient; bars, SEM. ****p < 0.0001, NS (non-significant) as determined by Student's t-test. [file Image_2.tif]

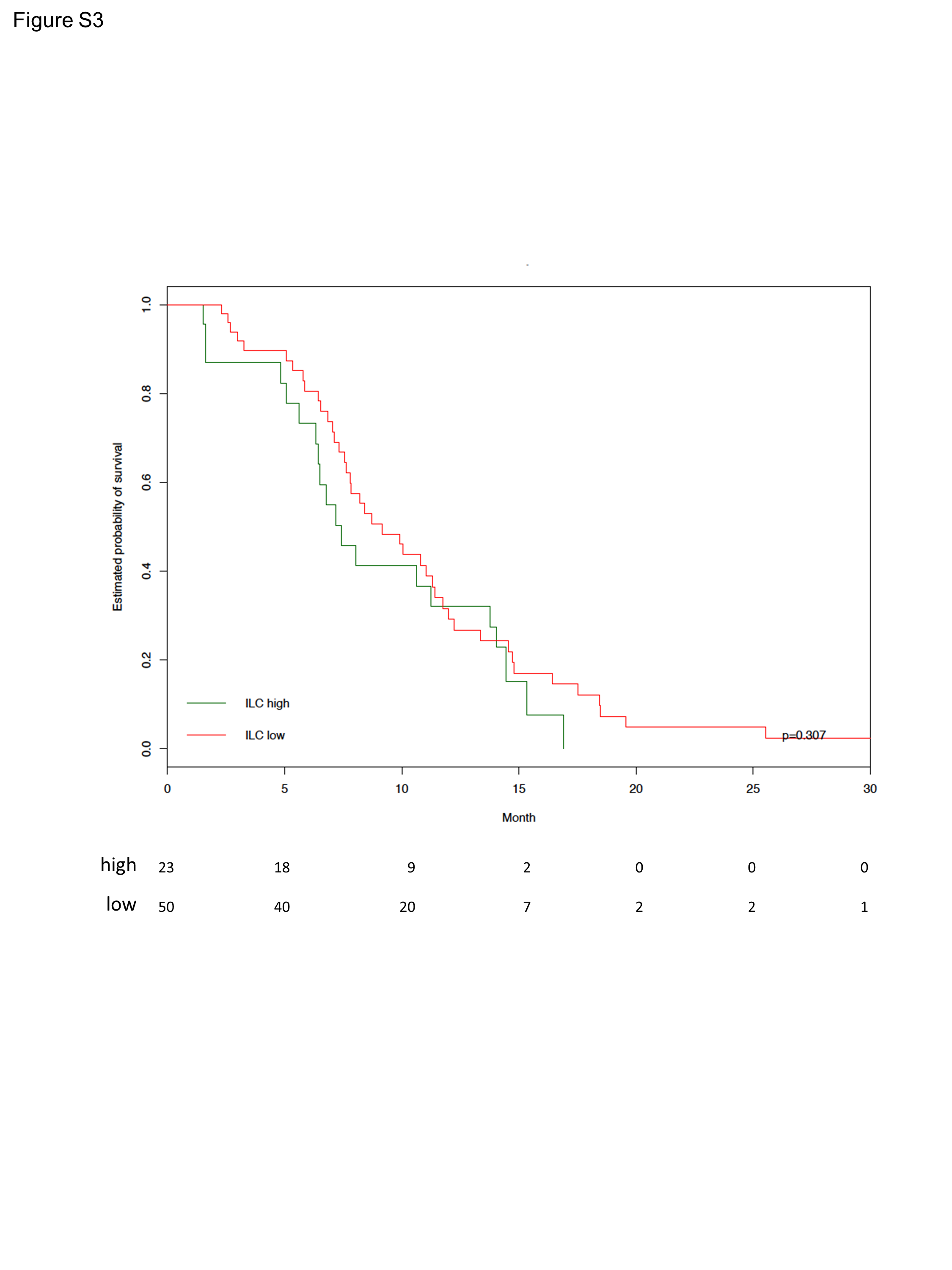

Supplement: Figure S3 — Progression-Free Survival Kaplan-Meier curves of chemotherapy-naïve metastatic colorectal cancer patients. Patients (n = 73) stratified by the frequency of total ILCs distributed into low + medium (n = 50) vs. high (n = 23) terciles. [file Image_3.tif]
