# Supplementary material for: Peripheral Innate Lymphoid Cells Are Increased in First Line Metastatic Colorectal Carcinoma Patients: A Negative Correlation With Th1 Immune Responses
Source: Front Immunol. 2019 Sep 6;10:2121. doi: 10.3389/fimmu.2019.02121 (PMC6742701; doi:10.3389/fimmu.2019.02121)
Supplement: Table S1 — Clinicobiological characteristics of CRC patients. The table summarizes the clinicobiological characteristics of CRC patients enrolled in the “Epitopes-CRC02” trial (NCT02817178) before (baseline) and after chemotherapy treatment (n = 96). [file Table_1.docx]

**Table S1: Clinicobiological characteristics of CRC patients.** The table summarizes the clinicobiological characteristics of CRC patients enrolled in the ‘Epitopes-CRC02’ trial (NCT02817178) before (baseline) and after chemotherapy treatment (n=96).

|  | **Patients** | **Patients at baseline** | **Patients after treatment** |
| --- | --- | --- | --- |
|  | n= 96 (%) | n= 86 (%) | n= 91 (%) |
| **Age - years** |  |  |  |
| <65 | n= 44 (46%) | 40 (47%) | 43 (48%) |
| >=65 | n= 51 (54%) | 45 (53%) | 47 (52%) |
| **Gender** |  |  |  |
| F | n= 33 (34%) | 31 (36%) | 31 (34%) |
| M | n= 63 (66%) | 55 (64%) | 60 (66%) |
| **Tumor location** |  |  |  |
| rectum | n= 27 (29%) | 22 (27%) | 26 (29%) |
| colon | n= 66 (71%) | 61 (73%) | 62 (71%) |
| **Microsatellites** |  |  |  |
| MSI | n= 9 (19%) | 8 (19%) | 9 (20%) |
| MSS | n= 38 (81%) | 35 (81%) | 36 (80%) |
| **RAS Status** |  |  |  |
| M | n= 43 (51%) | 37 (49%) | 41 (52%) |
| WT | n= 41 (49%) | 39 (51%) | 38 (48%) |
| **BRAF Status** |  |  |  |
| M | n= 9 (11%) | 9 (12%) | 9 (11%) |
| WT | n= 75 (89%) | 67 (88%) | 70 (89%) |
| **Time of metastasis** |  |  |  |
| Metachronous | n= 18 (19%) | 15 (18%) | 18 (20%) |
| Synchronous | n= 75 (81%) | 68 (82%) | 70 (80%) |
| **Metastatic localization** |  |  |  |
| Extra hepatic | n= 20 (21%) | 18 (22%) | 20 (23%) |
| Hepatic and other | n= 28 (30%) | 23 (28%) | 27 (31%) |
| Hepatic only | n= 45 (49%) | 42 (50%) | 41 (46%) |
| **Lymphocyte count** |  |  |  |
| <1000 | n= 11 (12%) | 10 (12.5%) | 11 (13%) |
| >=1000 | n= 79 (88%) | 70 (87.5%) | 74 (87%) |
| **ACE** |  |  |  |
| <20 | n= 36 (45%) | 31 (44%) | 34 (45%) |
| >=20 | n= 44 (65%) | 40 (56%) | 42 (55%) |
